# Analysis of research trends (2014-2023) on oxidative stress and male fertility based on bibliometrics and knowledge graphs

**DOI:** 10.3389/fendo.2024.1326402

**Published:** 2024-01-23

**Authors:** Chao Du, Yuexin Yu, Xinyue Fan

**Affiliations:** ^1^ Department of Reproductive Medicine, General Hospital of Northern Theater Command, Shenyang, Liaoning, China; ^2^ Department of Histology and Embryology, School of Basic Medicine, China Medical University, Shenyang, Liaoning, China; ^3^ Student Affairs Department of Shengjing Hospital, China Medical University, Shenyang, Liaoning, China

**Keywords:** oxidative stress, male fertility, bibliometrics, antioxidants, sperm DNA damage

## Abstract

**Background:**

Oxidative stress (OS) is considered one of the major factors affecting male fertility, and research in this field has seen constant growth year by year. Currently, around 700 relevant papers are published each year, with a trend of further growth. Therefore, this study systematically summarizes the literature published in the last decade from a bibliometric perspective, revealing the dynamic development of the field, identifying research hotspots, analyzing future trends, and providing reference for further research.

**Methods:**

Relevant literature on oxidative stress and male fertility was retrieved from the Web of Science Core Collection (WoSCC) database, covering the timespan from 2014 to 2023 and including two types, articles and reviews. CiteSpace and VOSviewer were used for bibliometric analysis, including cluster analysis, co-occurrence analysis, co-citation analysis, and burst analysis of countries/regions, institutions, journals, authors, references, and keywords.

**Results:**

This paper studied a total of 5,301 papers involving 107 countries/regions, with China having the highest number of publications (898 papers) and the United States having the highest centrality (0.62). Burst analysis of journal citations revealed the emergence of many new journals (e.g., *Antioxidants-Basel*, *Front Endocrinol*) after 2021, indicating continuous expansion and development in this field. Cluster analysis of co-cited references and co-occurring keywords divided the research into areas such as oxidative stress and male infertility, oxidative stress level detection, and antioxidants. The keywords associated with research hotspots shifted from oxidative stress detection, sperm DNA damage, apoptosis, and redox potential to DNA methylation, embryonic development, infection, polyunsaturated fatty acids, and antioxidants.

**Conclusion:**

Bibliometric methods provide an intuitive reflection of the development process in the field of oxidative stress and male fertility, as well as the analysis of research hotspots in different periods. Research on oxidative stress and embryonic development, as well as antioxidant health management, may become hotspots in future research.

## 1 Introduction

Infertility is defined as the inability of a couple to conceive after one year of unprotected sexual intercourse (1). Globally, approximately 8% to 12% of reproductive-age couples experience infertility issues, with male-related factors accounting for about 50% of all cases (2). Research has shown that the average sperm count and sperm density among adult males worldwide have declined by 60% and 52%, respectively, over the past 40 years, with a continued downward trend (3). Current research suggests that various factors may contribute to the decline in male fertility, and one of the primary mechanisms is the imbalance between oxidative stress and antioxidant capacity (4). Studies have confirmed that the antioxidant capacity of semen is generally reduced in infertile males (5). Oxidative stress refers to a state in which there is an imbalance between reactive oxygen species (ROS) and antioxidants in the body. While a small amount of ROS is necessary for normal physiological processes in the body, such as sperm capacitation and the acrosome reaction (6), an excess of ROS can cause damage to the body, including DNA damage, changes in protein structure, abnormal hormone levels, lipid peroxidation (LPO), and even epigenetic changes (7). It is now believed that the sources of ROS in seminal plasma mainly include immature sperm and white blood cells. Immature sperm refer to sperm that contain an excess of cytoplasmic residues in the middle part. On the one hand, the excess cytoplasmic disability has glucose-6-phosphate dehydrogenase (G6PD), which increases the content of nicotinamide adenine dinucleotide phosphate (NADPH), promoting the generation of ROS; sperm, on the other hand, contain a large number of mitochondria in the middle, which provide energy for movement. The ROS produced by mitochondria can cause damage to their own membranes, further increasing ROS levels (8). In addition to this, positive peroxidase leukocytes are able to produce ROS 1,000 times higher than normal sperm (9). Studies have shown that in addition to internal factors, lifestyle, environmental exposure and psychological factors can also affect the level of oxidative stress in the body (10–12).

**Figure 1:**
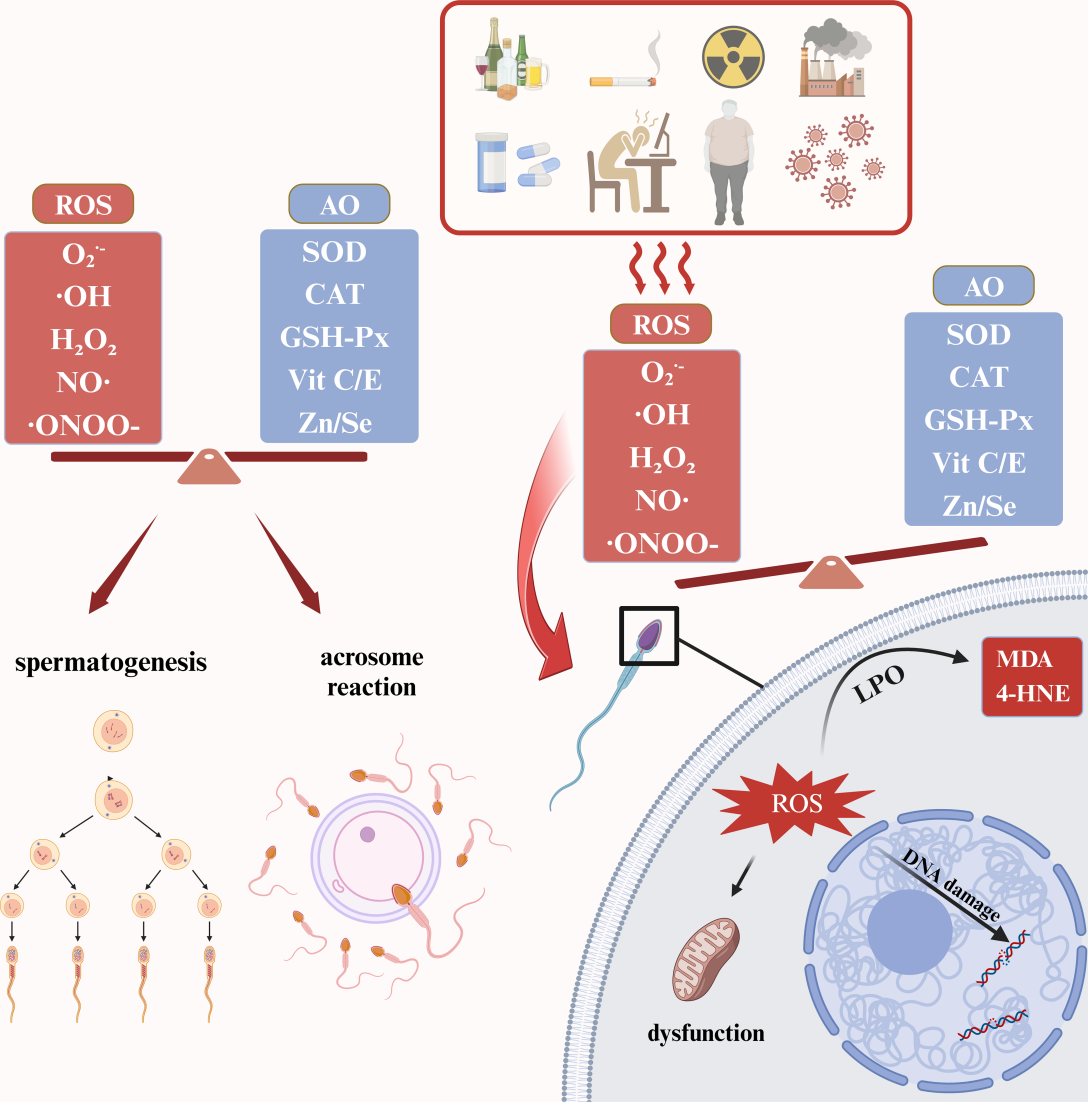
Physiological and pathological roles of ROS in male reproductive system. ROS, reactive oxygen species; AO, antioxidants; SOD, superoxide dismutase; CAT, catalase; GSH-Px, Glutathione peroxidase; MDA, malondialdehyde; 4-HNE, 4-hydroxynonenol; LPO, lipid peroxidation. The figure was produced with BioRender.

Under normal physiological conditions, oxidative stress is involved in spermatogenesis, maturation, capacitation and acrosome reaction. First, a variety of humoral factors are involved in the development and maturation of sperm. At this time, ROS affects spermatogenesis by regulating the receptor-tyrosine kinase (RTK) mediated pathway (13), while ROS contributes to the formation of protamine disulfide bonds and promotes DNA stability (14). When sperm are in the female reproductive tract, ROS drives sperm capacitation by activating the cAMP/PKA phosphorylation pathway. When a sperm meets an egg, ROS promotes an acrosome reaction by increasing the sperm’s affinity for the IP3 ligand to facilitate fertilization (13). The above physiological processes require a delicate balance of oxidation and antioxidant. Once the oxidation is intensified, it will lead to a series of adverse effects. For one thing, ROS causes the cell membrane of sperm to peroxidize. This can lead to the increase of cytotoxic LPO byproducts such as malondialdehyde (MDA) and 4-hydroxynonenol (4-HNE), impair membrane fluidity, and increase membrane ion permeability. For another, ROS can cause damage to mitochondrial and nuclear DNA and accelerate the process of apoptosis (15). In addition, it has been shown that ROS can increase the tyrosine nitration and S-glutathionylation of sperm, thereby obstructing sperm capacitation and leading to impaired sperm function (16) (**Figure 1**).

Due to the close connection between oxidative stress and male fertility, there has been an increasing amount of research in this field in recent years. Some articles have conducted related reviews (17, 18), but there is a lack of interpretation of research trends in this field and predictions for future development. Bibliometrics is a quantitative statistical analysis method used to analyze and assess research hotspots and trends (19). Using bibliometric methods, researchers can quickly understand the development process, collaborative relationships, and research hotspots in a particular field and predict future trends. This approach has been applied in various fields, including basic medicine, surgery, and psychology (20–22). This study utilized Citespace software developed by Chen Chaomei’s team (23) and VOSviewer software developed by Van Eck et al. (24) for a systematic analysis of literature related to oxidative stress and male fertility in the past decade. This analysis aims to help researchers better grasp the research status in this field and overall trends, and provide a reference for future research.

## 2 Materials and methods

### 2.1 Data source and search

The literature included in this study was sourced from the Web of Science Core Collection (WoSCC) database, as it is one of the most systematic and authoritative databases, which is widely recognized for its effectiveness in bibliometric research (25, 26). The search was conducted on June 22, 2023, using the following search query: (TS= “Spermatozoa” OR “Sperm” OR “male fertility” OR “male sterility” OR “male reproduction”) AND (TS= “Reactive Oxygen Species” OR “Oxidative stress”). The following search limitations were applied:

(1) Timespan: 2014 to 2023;
(2) Document type: articles AND reviews;
(3) No species restriction;
(4) Language: English.

After screening, a total of 5,319 articles were retrieved. For articles meeting these inclusion criteria, all records, including titles, authors, abstracts, keywords, and references, were exported as plain text files and saved as download_txt files. Then, they were imported into CiteSpace 5.7.R5, and after deduplication, 5,301 articles remained for further analysis.

### 2.2 Data analysis and visualization

The researchers used CiteSpace 5.7.R5 to analyze countries, institutions, authors, and citations. CiteSpace allows for data visualization by constructing citation networks and co-occurrence networks, presenting the structure, patterns, and distribution of scientific knowledge in a specific discipline or field (27). In this study, the nodes in the network were represented using a yearly ring method, where the overall size of nodes reflects their citation or occurrence frequency, the width of the yearly ring represents the number of papers published in a given year, and the connections between nodes indicate shared citations or co-occurrence frequency with other nodes. The specific parameters used in this study were: Time slicing: Jan. 2014 to Dec. 2023, Years per slice: 1, Term source: Title, Abstract, Author Keywords, and Keyword Plus, Node type: Author, Institution, Country, Keyword, Reference, Cited Author, and Cited Journal, Link strength: Cosine, Selection Criteria: g-index, k = 20, Pruning: Pathfinder, Pruning the merged network, Visualization: Cluster View-Statistics, Show Merged Network. Additionally, keyword clustering analysis was conducted using VOSviewer 1.6.19, with parameters set to default values. The colors of nodes represent their membership in specific clusters, with different colors indicating different clusters, allowing for the discovery of structural distributions of research hotspots.

## 3 Results

### 3.1 Annual publications

**Figure 2:**
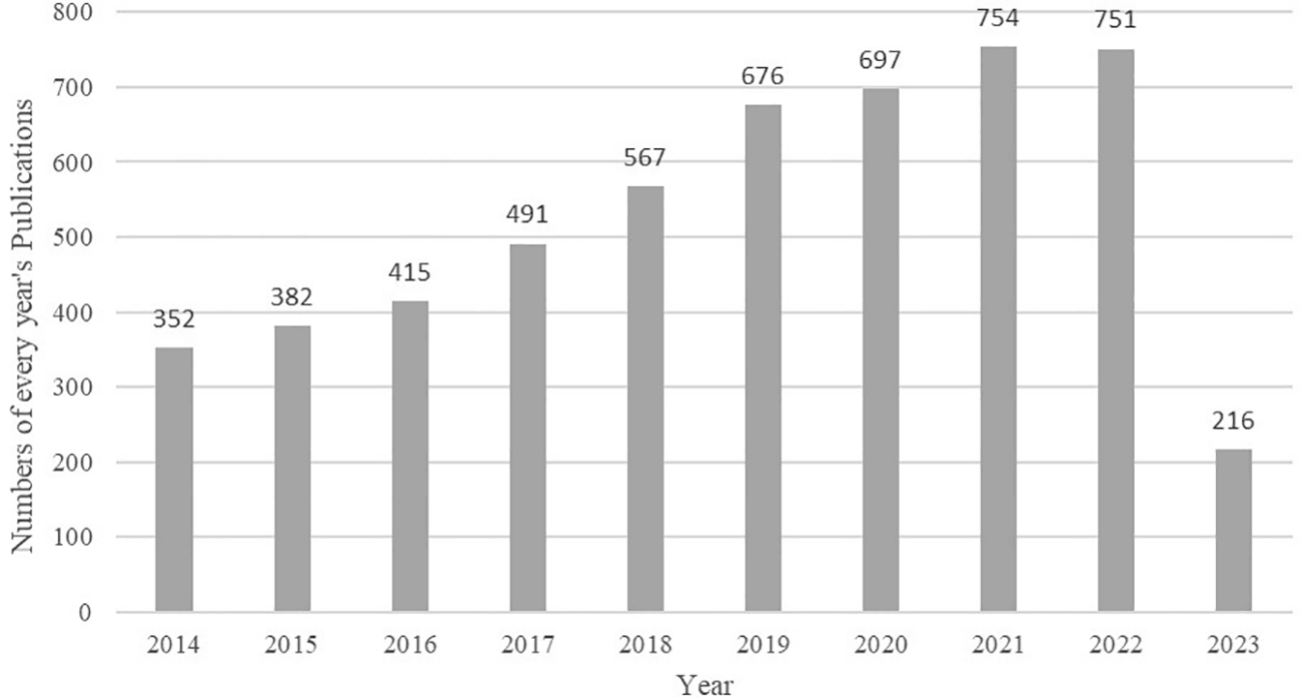
The number of OS and male fertility research publications from 2014 to 2023.

A total of 5,301 articles were included in the analysis. The annual number of publications increased from 352 in 2014 to 751 in 2022, as shown in **Figure 2**. Through statistical analysis, it was found that in the last decade, the author with the highest number of publications was *Ashok Agarwal*, who published 66 papers. The country/region with the most publications was *Peoples R China*, where 898 papers were published, and the institution with the most publications was *Univ Sao Paulo*, publishing 115 papers. The centrality of authors, institutions, and countries/regions was generally low, indicating a need for increased collaboration in the field. The top 5 authors, countries/regions, and institutions in terms of publications in the last decade are detailed in **Table 1**.

**Table 1:** Top 5 authors, countries/regions, and institutions by number of publications (2014-2023).

Ranking	Author	Number of Publications	Centrality	Year
1	Ashok Agarwal	66	0.01	2014
2	Eva Tvrda	28	<0.01	2015
3	Mohsen Sharafi	26	<0.01	2017
4	Muhammad Umar Ijaz	21	0.01	2016
5	Marcilio Nichi	21	0.01	2019
Ranking	Country/Region	Number of Publications	Centrality	Year
1	Peoples R China	898	0.30	2014
2	Iran	553	0.18	2014
3	USA	442	0.62	2014
4	Egypt	333	0.11	2014
5	Turkey	302	0.08	2014
Ranking	Institution	Number of Publications	Centrality	Year
1	Univ Sao Paulo	115	0.03	2014
2	Cairo Univ	114	0.02	2014
3	ACECR	89	0.21	2014
4	Northwest A&F Univ	82	0.02	2015
5	Islamic Azad Univ	80	0.13	2014

### 3.2 Journals and co-cited academic journals analysis

**Figure 3:**
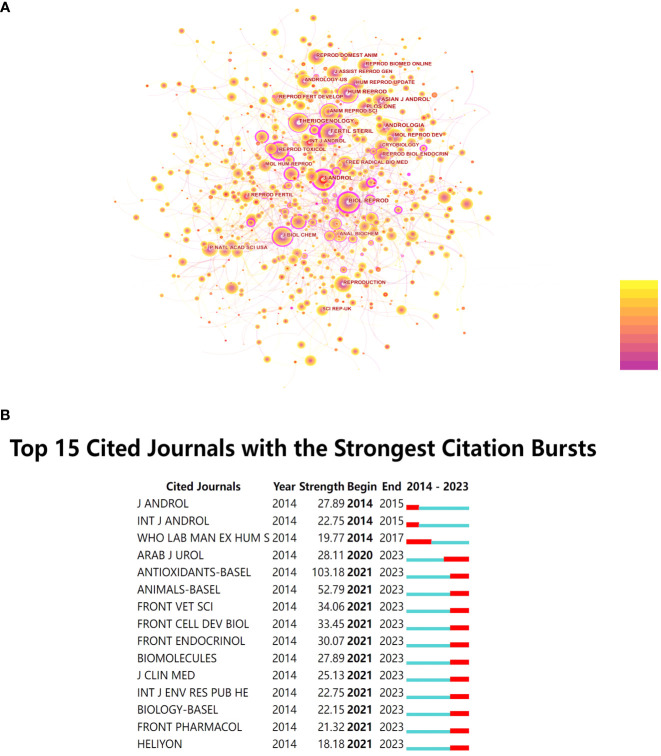
**(A)** Co-citation Network Diagram of Journals. **(B)** Top 15 Cited Journals with the Strongest Citation Bursts.

After conducting co-citation analysis on all journals, the researchers observed that the number of nodes increased from 401 in 2014 to 690 in 2022. Additionally, the number of connections grew from 1,203 to 2,070 during this period. These findings indicate that research in this field has garnered widespread attention in recent years, with increased mutual referencing and a trend of further development. From **Figure 3A**, it is evident that the journal with the highest number of citations and occupying a central position in this field is “*Fertil Steril*” with 2,894 citations and a centrality score of 0.62. Detailed information on the top 10 most frequently cited journals is provided in **Table 2**.

**Table 2:** The top 10 co-cited journals ranked by number of citations.

Co-cited journals	Citations	Country	Centrality	2022 IF	2022 JCR partition
*Fertil Steril*	2894	US	0.62	6.70	Q1
*Biol Reprod*	2633	US	0.41	3.60	Q1
*J Androl*	2603	US	0.42	4.50	Q1
*Andrologia*	2584	GER	0.07	2.40	Q4
*Hum Reprod*	2490	UK	0.13	6.10	Q1
*Theriogenology*	2219	US	0.12	2.80	Q4
*Plos One*	2018	US	<0.01	3.70	Q2
*Asian J Androl*	1871	CHN	0.07	2.90	Q2
*Anim Reprod SCI*	1723	NL	0.11	2.20	Q2
*J Biol Chem*	1630	US	0.22	4.8	Q1

Furthermore, this study conducted burst detection analysis on the cited journals. When a journal experiences a sudden increase in citation frequency within a specific period, it is marked in red. As shown in **Figure 3B**, starting from 2021, many journals have exhibited a sudden increase in co-citation frequency, and this trend has continued. Among them, “*Antioxidants-Basel*” stands out with a burst intensity of 103.18.

### 3.3 Analysis of co-cited references

**Figure 4:**
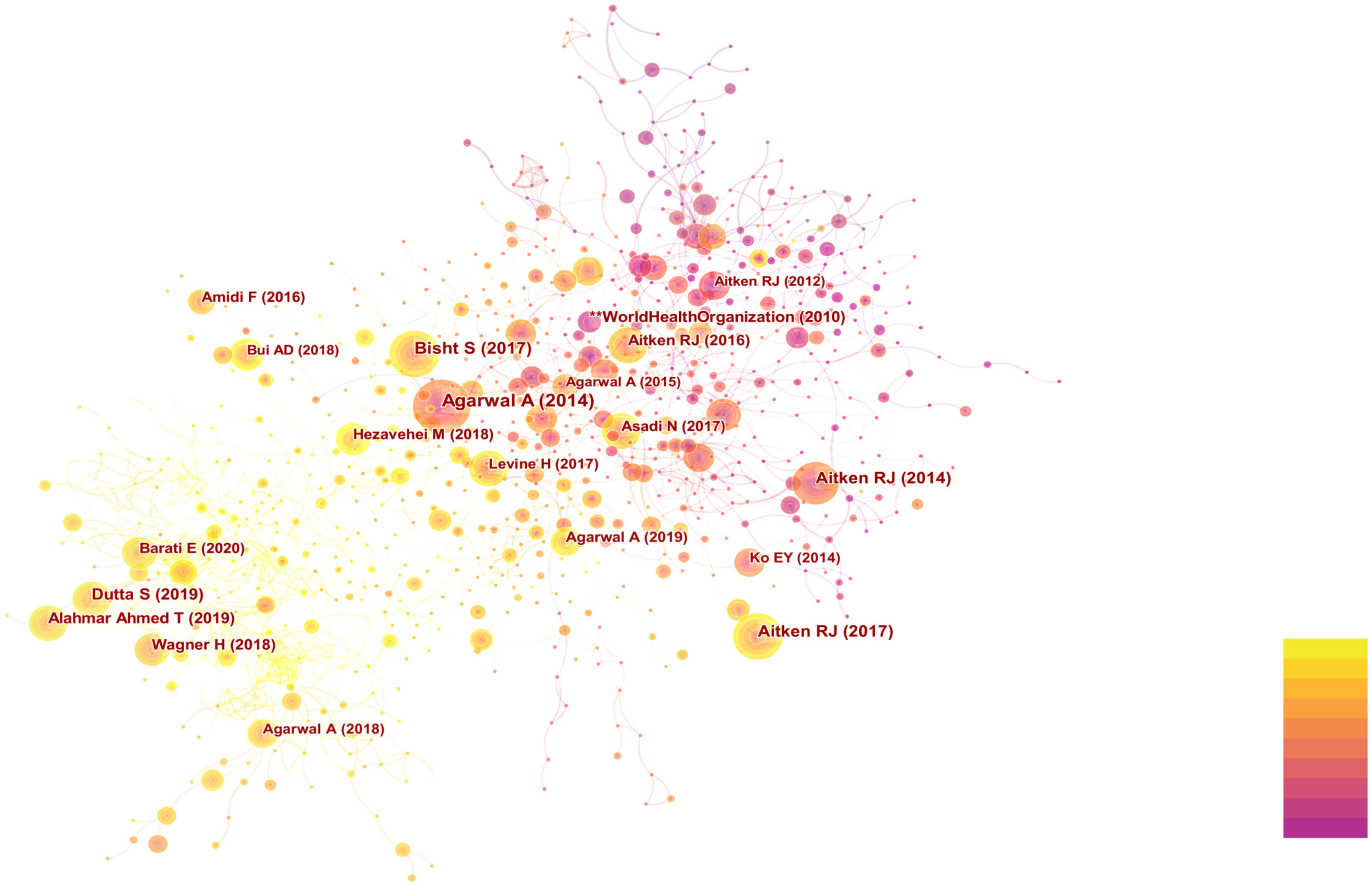
Co-citation network diagram of references related to OS and male fertility.

The researchers utilized CiteSpace to conduct co-citation analysis on the top 10% most frequently cited references within each time period. Co-citation relationships are established when two references are both cited by a third reference. References with a high co-citation frequency indicate their significant influence within their respective field of research, often referred to as core references. The co-citation network comprised a total of 1,015 nodes and 4,456 connections. **Figure 4** displays the authors and publication years of articles cited more than 50 times. The top 10 most frequently cited references are listed in **Table 3** (17, 18, 28–35).

**Table 3:** Top 10 co-cited references in the research on OS and male fertility.

Publication Year	First Author	Title	Journal	Citations	Centrality
2014	Agarwal A^[28]^	Effect of Oxidative Stress on Male Reproduction	*World J Mens Health*	187	<0.01
2017	Bisht S^[29]^	Oxidative stress and male infertility	*Nat Rev Urol*	126	0.02
2017	Aitken RJ^[30]^	Reactive oxygen species as mediators of sperm capacitation and pathological damage	*Mol Reprod Dev*	111	<0.01
2014	Aitken RJ^[31]^	Oxidative stress and male reproductive health	*Asian J Androl*	105	<0.01
2019	Dutta S^[17]^	Oxidative stress and sperm function: A systematic review on evaluation and management	*Arab J Urol*	91	0.01
2019	Alahmar Ahmed T^[32]^	Role of Oxidative Stress in Male Infertility:An Updated Review	*J Hum Reprod SCI*	82	<0.01
2010	World Health Organization^[33]^	WHO laboratory manual for the examination and processing of human semen. 5th ed.	*Lab Man Ex Proc Hum*	80	<0.01
2016	Aitken RJ^[34]^	Causes and consequences of oxidative stress in spermatozoa	*Reprod Fert Develop*	77	<0.01
2018	Wagner H^[35]^	Role of reactive oxygen species in male infertility: An updated review of literature	*Arab J Urol*	69	<0.01
2017	Asadi N^[36]^	The Impact of Oxidative Stress on Testicular Function and the Role of Antioxidants in Improving it: A Review	*J Clin Diagn Res*	67	0.01

**Figure 5:**
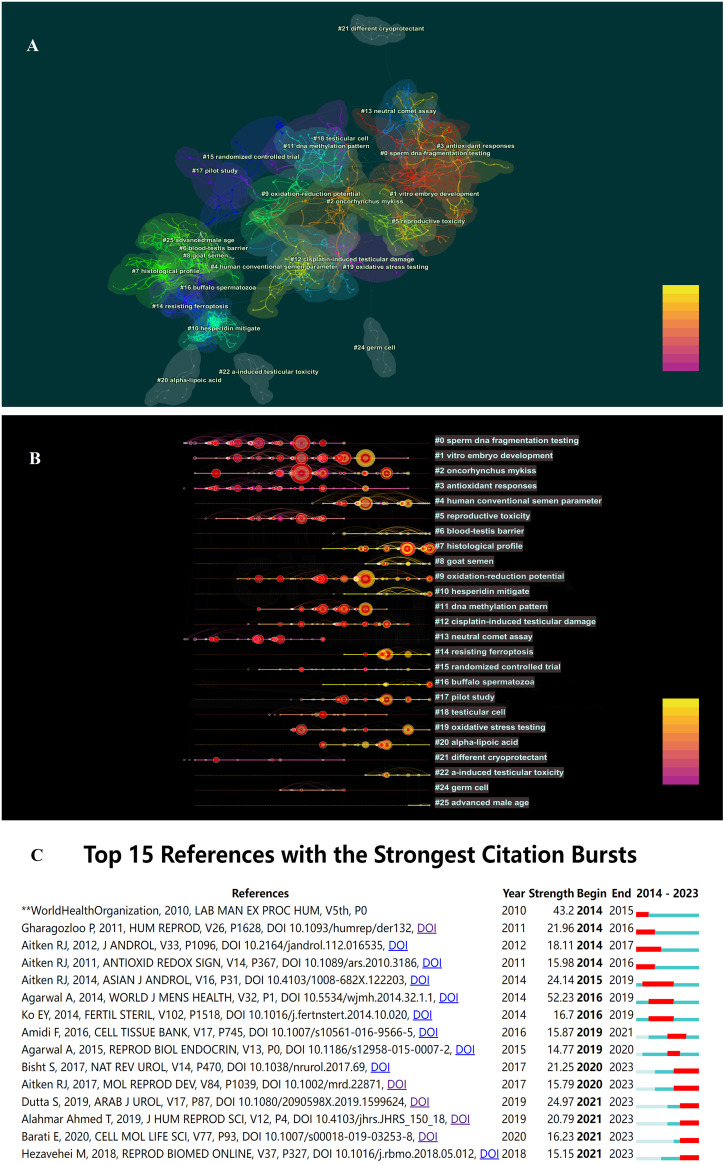
**(A)** The co-citation clusters of references related to OS and male fertility. **(B)** The timeline view of references related to OS and male fertility. **(C)** The co-citation clusters of references related to OS and male fertility.

Upon conducting cluster analysis on references cited more than 5 times, this study identified a total of 64 clusters, with clustering metrics showing Modularity Q = 0.7964 and Weighted Means Silhouette = 0.9141, indicating significant modularity and reasonable clustering results. Detailed clustering results are presented in **Figure 5A**. The fish-eye view (**Figure 5B**) illustrates the temporal distribution of cited references in different categories, with nodes positioned further to the right representing references cited more recently. Additionally, citation burst detection (**Figure 5C**) revealed bursts in citations each year from 2014 to 2023, indicating a sustained trend of development in this field of research.

It is noteworthy that over the past decade, the most frequently cited reference with the highest burst intensity was a review article by *Agarwal A* et al. (28), titled “*Effect of Oxidative Stress on Male Reproduction*,” published in *World J Mens Health* in 2014, cited 187 times with a burst intensity of 52.23.

### 3.4 Hotspots and Frontiers analysis

**Figure 6:**
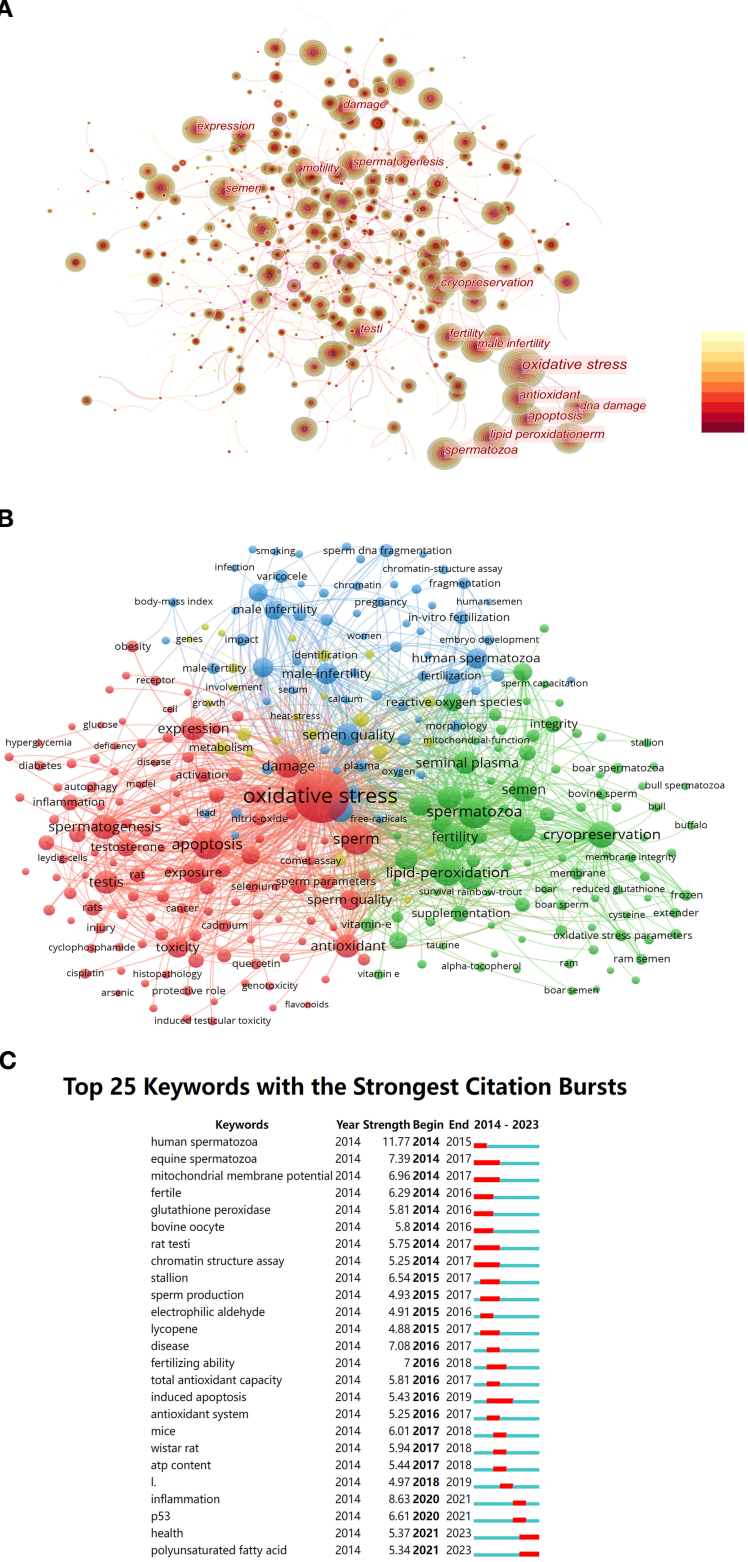
**(A)** Co-occurrence network diagram of key words related to OS and male fertility. **(B)** The clusters of keywords related to OS and male fertility. **(C)** Top 25 keywords with strongest citation bursts.

Based on a co-occurrence analysis of keywords using CiteSpace, the researchers found a total of 678 nodes and 6,781 connections in the keyword co-occurrence network. The most frequently occurring keywords were “Oxidative stress” (3766), “spermatozoa” (1068), “sperm” (914), “apoptosis” (876), “antioxidant” (863), “lipid peroxidation” (847), “cryopreservation” (678), “DNA damage” (655), and so on. These keywords reflect the main research directions and content in this field. In **Figure 6A**, keywords that appeared more than 500 times are highlighted.

Furthermore, this study conducted cluster analysis of keywords using VOSviewer for keywords that co-occurred more than 30 times. We identified four clusters: Cluster 1 in red (124 keywords), Cluster 2 in green (94 keywords), Cluster 3 in blue (67 keywords), and Cluster 4 in yellow (24 keywords). Details of the keywords within each cluster are shown in **Figure 6B**.

It is evident that Cluster 1 primarily includes keywords related to oxidative stress, sperm development, and apoptosis. Cluster 2 contains keywords related to sperm cryopreservation and cryoprotectants. Cluster 3 focuses on keywords related to male infertility, such as “male infertility,” “smoking,” “varicocele,” and “sperm DNA fragmentation.” Cluster 4 includes fewer keywords with lower occurrence frequencies, primarily related to such terms as “metabolism” and “involvement.”

Furthermore, burst detection analysis in **Figure 6C** revealed the existence of burst keywords nearly every year from 2014 to the present. These keywords have evolved from terms like “human spermatozoa,” “mitochondrial membrane potential,” and “glutathione peroxidase” in 2014 to terms like “inflammation,” “P53,” “health,” and “polyunsaturated fatty acid” after 2020.

## 4 Discussion

Oxidative stress plays a role in various physiological processes in the body, but research has also confirmed its association with a range of pathological phenomena when it becomes imbalanced. Several factors contribute to the decline in male fertility by exacerbating oxidative stress within the body. These factors include endogenous factors such as inflammation, smoking, varicocele, and obesity (36–39), as well as exogenous factors like radiation, phthalates (PAEs), and air pollution (40–42). As the impact of oxidative stress on male fertility garners increasing attention, it becomes crucial to summarize the research status in this field and make reasonable predictions about future trends.

In this study, the researchers employed bibliometric methods to analyze relevant literature from the past decade, encompassing a total of 5301 articles. A glance at the publication volume curve reveals a consistent year-on-year increase in publications in this field. The research spans across 74 countries or regions and involves 580 institutions. Among these, China has contributed the highest number of articles, while the United States holds the highest centrality. Co-citation analysis of journals has uncovered the formation of a core group of journals in the field, with “*Fertil Steril*” and “*Biol Reprod*” at the forefront. Most of these journals are categorized in Q1, signifying substantial recognition among researchers. Furthermore, the analysis of journal bursts has revealed the emergence of a new wave of highly cited journals, such as “*Antioxidants-Basel*” and “*Front Endocrinol*,” which not only cover areas like cell biology and endocrinology but also extend into biochemistry, molecular biology, and veterinary science. This expansion indicates a growing and rapidly developing trend in the field.

In addition to analyzing the basic characteristics of publications, authors, countries or regions, and journals, this study takes full advantage of bibliometric methods to analyze citations and keywords. This analysis provides an intuitive representation of the research content related to oxidative stress and male fertility over the past decade and makes predictions regarding potential research hotspots in the future. In the following sections, this paper will delve further into the discussion, focusing on highly co-occurring and burst keywords.

### 4.1 Oxidative stress and male infertility

Cellular oxidative stress can result from an increase in the production of reactive oxygen species (ROS), such as hydroxyl radicals (OH), superoxide anions (O2−), and hydrogen peroxide (H2O2) (43). It can also occur due to a decrease in antioxidant defenses. The antioxidant system includes enzymatic factors like superoxide dismutase, catalase, and glutathione peroxidase, as well as non-enzymatic factors like glutathione, N-acetylcysteine, vitamin E, coenzyme Q10, carnitines, myo-inositol, and lycopene, along with trace elements such as selenium, zinc, and copper (44–46).

Sperm cells are among the first to exhibit susceptibility to oxidative damage. This susceptibility arises from several factors. Sperm cells possess abundant mitochondria to provide energy for their motility, and mitochondria are considered a primary source of ROS. ROS, by affecting electron transfer in the respiratory chain, activate intracellular signaling cascades, leading to increased ROS production within mitochondria (47). Sperm cells also have plasma membranes rich in polyunsaturated fatty acids (PUFAs), making them vulnerable to oxidative stress. When the sperm plasma membrane undergoes lipid peroxidation (LPO), it disrupts membrane permeability, resulting in ATP efflux and impairing flagellar motility, ultimately affecting sperm motility and fertilization potential (15).

The impact of oxidative stress on male fertility is further evidenced by its role in exacerbating sperm DNA damage (48). Existing research indicates that an increase in sperm DNA damage can affect the developmental potential of embryos post-fertilization, leading to an elevated risk of miscarriage and an increased likelihood of birth defects in offspring (49, 50). Studies have shown that sperm obtained through testicular sperm aspiration (TESA) exhibit significantly lower DNA fragmentation than ejaculated sperm and have better outcomes in intracytoplasmic sperm injection (ICSI) treatments (51). Currently known sperm DNA breaks include single strand DNA breaks (SSBs) and double strand DNA breaks (DSBs), which cause different clinical reproductive effects (52). For one thing, SSBs is linked to oxidative stress and is widespread in all regions of the genome in the form of multiple breakpoints that can lead to prolonged conception or infertility (53). For another, DSBs are mainly confined to the sperm nuclear stroma and may be associated with inadequate DNA repair during meiosis, resulting in an increased risk of miscarriage, decreased embryo quality, and increased risk of implantation failure during ICSI cycles (54). Studies have shown that oocytes can repair DNA damage of sperm, mostly through base excision repair (BER). But this repair ability decreases with oocyte aging and associated decent in female gamete’s quality. A previous study in mice showed that this might be due to the significant decrease in the expression of some key genes of DSB repair pathway, such as *Brca1* and *Brca2*, in MII oocytes with age (55).

### 4.2 Oxidative stress level assessment

With advancements in technology, assessing sperm quality solely based on traditional seminal parameters evidently falls short of meeting contemporary clinical demands. Oxidative stress levels are now regarded as a novel indicator for evaluating sperm quality. In 2019, literature introduced the concept of “Male Oxidative Stress Infertility” (56). This concept utilizes the Male Infertility Oxidative System (MiOXSYS) to measure the oxidation-reduction potential (ORP) of the semen as a means to assess oxidative stress levels. The advantage of this method is that it can detect oxidative stress levels in real time. The sample may be either semen or seminal plasma. This method needs short time and is easy to operate, but its disadvantage is that the detection result is affected by the viscosity of the sample. Henkel et al. conducted a prospective observational study demonstrating that seminal ORP values detected by MiOXSYS have significant predictive power for fertilization rate, blastocyst development rate, implantation/clinical pregnancy, and live birth rate during ICSI cycles. Based on the assisted reproduction results, the relevant calculated cut-off values for seminal ORP predicting good fertilization (>80%), blastocyst formation (>60%), implantation/clinical pregnancy and live birth were ≤0.709, ≤0.530, ≤0.465 and ≤0.393 mV/10^6^ sperm/ml, respectively, with an average value of 0.51 mV/10^6^ sperm/ml (57). Interestingly, these figures were significantly lower than the threshold ORP of 1.34 mV/10^6^ sperm/ml for men with abnormal and normal semen parameters in previous studies (58).

In addition to seminal ORP measurement, there are alternative methods for oxidative stress assessment, including total antioxidant capacity (TAC), superoxide dismutase (SOD), and malondialdehyde (MDA) assays. However, it remains challenging to incorporate these tests into routine use due to their cost, complexity, time sensitivity, and the potential requirement for sophisticated equipment, substantial and well-handled sample volumes, as well as extensive technical training (59). Apart from the mentioned indicators, the sperm DNA fragment index (DFI) is also considered an indirect marker that can provide insights into oxidative stress (60). There are a variety of methods to test sperm DNA fragments, such as sperm chromatin structure assay (SCSA), sperm chromatin dispersion (SCD) assay, terminal deoxynucleotide transferase-mediated deoxyuridine triphosphate notch end labeling (TUNEL) assay, and single cell gel electrophoresis (Comet) assay (61). The advantage of TUNEL, SCSA, and SCD lies in their highly standardized protocol, but TUNEL and SCSA require flow cytometer. Comet assay is able to differentially detect MAR-region double-strand breaks, but technique and analysis are not standardized between laboratories. In earlier studies, the cut-off value for DFI was usually set to DFI = 27% or 30% (62, 63), but some studies in recent years have used a lower cut-off value, such as DFI = 20% (64–66). The author believes that it is difficult to determine the optimal ORP or DFI cut-off value due to different clinical scenarios and study objects. In clinical work, doctors still need to take this as a reference to propose a more personalized treatment plan. In general, while oxidative stress assessment is considered an emerging marker for evaluating male fertility, there remains a lack of scientifically practical detection methods and diagnostic standards. Further research is needed to establish these measures for the diagnosis and management of high oxidative stress levels.

### 4.3 Semen cryopreservation and oxidative stress

Currently, sperm cryopreservation has become one of the essential techniques in assisted reproduction and fertility preservation. Research reports have indicated the adverse effects of freezing technology on sperm vitality, fertilization capacity, and DNA integrity, with these effects being particularly noticeable in infertile men or low-quality sperm samples (67). Oxidative stress (OS) is considered one of the primary reasons for the decreased sperm survival rate and capabilities following ultra-low-temperature storage. Studies have confirmed that during the process of freezing and thawing, changes occur in the fluidity of the sperm mitochondrial membrane, leading to mitochondrial dysfunction and an increased release of reactive oxygen species (ROS), ultimately causing single-strand and double-strand DNA breaks (68).

In recent years, researchers have conducted detailed studies on the mechanisms of cryopreservation-induced damage and the effects of antioxidant additives using animal sperm. For example, studies involving the cryopreservation of ram semen have revealed that freezing leads to the opening of the mitochondrial permeability transition pore (mPTP), resulting in increased permeability of the inner mitochondrial membrane, which leads to an elevation in calcium (Ca2+) and ROS release (69). Compounds such as MitoQ and L-carnitine have been found to mitigate cryopreservation-induced sperm damage by specifically targeting mitochondrial oxidative stress (70, 71). Supplementation of melatonin in the cryopreservation medium for ram sperm has been shown to inhibit the opening of mPTP, thereby improving crucial aspects of mitochondrial oxidative phosphorylation kinase (OXPHOS kinase), oxygen consumption, ATP synthesis, as well as sperm vitality, motility, and kinetics. Interestingly, melatonin-treated cryopreserved sperm have demonstrated higher rates of blastocyst formation and pregnancy following *in vitro* fertilization and artificial insemination (72).

### 4.4 Antioxidant therapy

Antioxidant therapy is considered one of the crucial strategies for improving sperm quality (73). Maintaining a healthy diet and lifestyle is the primary approach to reducing oxidative stress within the body (74). Additionally, treatments such as surgery for varicocele (75) and anti-inflammatory therapies (76) have been shown through research to effectively enhance semen ROS (Reactive Oxygen Species) levels. Furthermore, supplementation with various antioxidants is beneficial for neutralizing ROS within the body. These include enzymes like superoxide dismutase (SOD), glutathione (GSH), and N-acetylcysteine (NAC), as well as non-enzymatic antioxidants like vitamin A, vitamin E, coenzyme Q10, and essential trace elements (77, 78). Researchers have also been exploring the use of plant extracts to improve oxidative stress treatment (79). Although the efficacy of many antioxidants has been demonstrated through studies, no consensus has been reached regarding the standardized dosages and durations of antioxidant intake. In addition, some studies have shown that exogenous antioxidant intake is not appropriate for all infertile men. Antioxidant paradox and reductive stress have been carefully elaborated, suggesting that excessive antioxidant intake may have adverse effects on male fertility (80, 81). Therefore, it is necessary to test the REDOX status of patients before proceeding with antioxidant therapy. The management of patients with such conditions remains a significant challenge for both researchers and clinical practitioners.

## 5 Conclusion

To sum up, this study is the first to systematically analyze the studies on oxidative stress and male fertility in the past 10 years using bibliometrics, and successfully establish a knowledge map in this field. From citation analysis and keyword analysis, we can find that people have a more comprehensive understanding of the mechanism of oxidative stress affecting male fertility. People no longer blindly pursue the benefits of antioxidants, but put forward the concept of the balance between oxidants and antioxidants, and are paying more attention to the impact of other factors such as inflammation; in the detection of oxidative stress level, a variety of direct or indirect indicators have emerged, such as TAC, LPO markers (MDA, 4-HNE, etc.), DFI and ORP. A large number of studies have confirmed their clinical value, and the operation method is becoming more and more simple and standardized, filling the gap of traditional semen index to evaluate male fertility. However, due to the differences in clinical scenarios and research objects, the cut-off values of the above indicators are still difficult to be determined, and RCT experiments with a larger sample size are needed for further verification. In the future, with the development of proteomics and metabolomics, more convincing indicators are expected to be found. In the treatment of oxidative stress, the use of antioxidants has changed from single drug to combination drug, and more attention has been paid to etiological treatment. In addition to traditional antioxidants such as vitamin E, research on herbal extracts is growing. How to develop a personalized antioxidant use plan based on the detection index of oxidative stress level in infertile men is an important problem that needs to be solved in the future.

However, there remain certain limitations in this study: 1) Due to the formatting requirements of Citespace, the analysis was conducted only on literature retrieved from WoSCC, and other databases such as PubMed and Scopus were not included. 2) The search was conducted until June 2023, which means that only a portion of 2023 research was available online, potentially limiting the ability of this paper to reflect the research trend of the entire year. Further research is needed to address these limitations.

## Data availability statement

The original contributions presented in the study are included in the article/supplementary material. Further inquiries can be directed to the corresponding author.

## Author contributions

CD: Data curation, Formal analysis, Methodology, Visualization, Writing – original draft, Writing – review & editing. YY: Formal analysis, Project administration, Resources, Supervision, Writing – review & editing. XF: Data curation, Formal analysis, Methodology, Visualization, Writing – original draft.
